# Guidance for Developers of Health Research Reporting Guidelines

**DOI:** 10.1371/journal.pmed.1000217

**Published:** 2010-02-16

**Authors:** David Moher, Kenneth F. Schulz, Iveta Simera, Douglas G. Altman

**Affiliations:** 1 Ottawa Methods Centre, Clinical Epidemiology Program, Ottawa Hospital Research Institute, Ottawa, Ontario, Canada; 2 Department of Epidemiology and Community Medicine, Faculty of Medicine, University of Ottawa, Ottawa, Ontario, Canada; 3 Family Health International, Research Triangle Park, North Carolina, United States of America; 4 Centre for Statistics in Medicine, University of Oxford, Oxford, United Kingdom

## Abstract

David Moher and colleagues from the EQUATOR network offer guidance and recommended steps for developing health research reporting guidelines.

## Introduction

Publishing health research is a thriving, and increasing, enterprise. On any given month about 63,000 new articles are indexed in PubMed, the United States National Library of Medicine's public access portal for health-related publications. However, the quality of reporting in most health care journals remains inadequate. Glasziou and colleagues [1] assessed descriptions of given treatments in 80 trials and systematic reviews for which summaries were published during one year (October 2005 to October 2006) in *Evidence-Based Medicine*, a journal that is aimed at physicians working in primary care and general medicine. Treatment descriptions were inadequate in 41 of the original published articles, which made their use in clinical practice difficult if not impossible to replicate. This is just one of numerous examples of a large and disturbing literature indicating the general failure in the quality of reporting health research [2]–[6]. Many publications lack clarity, transparency, and completeness in how the authors actually carried out their research.

Inadequate reporting is problematic for several reasons. If authors do not provide sufficient details concerning the conduct of their study, readers are left with an incomplete picture of what was done. As such, they are not able to judge the reliability of the results and interpret them. There are also ethical and moral reasons for reporting research adequately [7].

The EQUATOR (Enhancing the QUAlity and Transparency Of health Research) Network is a new international initiative seeking to improve the quality of scientific publications by promoting transparent and accurate reporting [8]. The Network (http://www.equator-network.org) provides resources and training relating to the reporting of health research and assists in the development, dissemination, and implementation of reporting guidelines. As part of its initial resource development, the Network's Web site contains a comprehensive and up-to-date database of reporting guidelines relevant to heath research. A recent systematic review of 81 reporting guidelines found their development was often inadequate [9].

Reporting guidelines need to be differentiated from other efforts that produce a checklist or other guidance not specific to reporting research. We propose here a working definition of a reporting guideline: *a checklist, flow diagram, or explicit text to guide authors in reporting a specific type of research, developed using explicit methodology*. Some reporting guidelines recommend a flow diagram so that authors can clearly report information about sequential stages of their research project. A consensus process [10] should be a crucial characteristic of developing a reporting guideline.

The main motivation for the development of reporting guidelines is to help researchers improve the completeness and transparency of their research reports and limit the number of poorly reported studies. However, reporting guidelines can be also used by peer reviewers and editors to strengthen manuscript review. And research funders can benefit from introducing reporting guidelines into the research application system [11]. Ensuring clear and complete reporting of funded research through the use of reporting guidelines should facilitate more efficient use of the new findings and bring better returns on research investments. There are enormous potential benefits of good reporting. However, despite the impressive recent upsurge in the number and range of reporting guidelines, the literature on how individual guidelines were developed remains sparse [12],[13] and there is no generic guidance on how to develop one.

In this paper we update and expand upon an earlier effort to outline a strategy for developing reporting guidelines that was published only in Spanish [14]. We recognize that there is no single best or correct approach. However, this paper benefits from our collective experiences of helping to develop more than ten reporting guidelines over the last 16 years, over which period these ideas have evolved considerably. If reporting guidelines are to be useful and more widely disseminated, they need to be developed using robust and widely accepted methodologies.

This strategy assumes the involvement of an executive group to facilitate the guideline development and the expectation of having a face-to-face meeting as part of the reporting guideline development. We propose 18 steps to occur in five phases, which are outlined in Table 1.

**Table 1:** Recommended steps for developing a health research reporting guideline.

Step	Item Number	Detail
**Initial steps**	1	Identify the need for a guideline
	1.1	Develop new guidance
	1.2	Extend existing guidance
	1.3	Implement existing guidance
	2	Review the literature
	2.1	Identify previous relevant guidance
	2.2	Seek relevant evidence on the quality of reporting in published research articles
	2.3	Identify key information related to the potential sources of bias in such studies
	3	Obtain funding for the guideline initiative
**Pre-meeting activities**	4	Identify participants
	5	Conduct a Delphi exercise
	6	Generate a list of items for consideration at the face-to-face meeting
	7^a^	Prepare for the face-to-face meeting
	7.1	Decide size and duration of the face-to-face meeting
	7.2	Develop meeting logistics
	7.3	Develop meeting agenda
	7.3.1	Consider presentations on relevant background topics, including summary of evidence
	7.3.2	Plan to share results of Delphi exercise, if done
	7.3.3	Invite session chairs
	7.4	Prepare materials to be sent to participants prior to meeting
	7.5	Arrange to record the meeting
**The face-to-face consensus meeting itself**	8^a^	Present and discuss results of pre-meeting activities and relevant evidence
	8.1^a^	Discuss the rationale for including items in the checklist
	8.2	Discuss the development of a flow diagram
	8.3^a^	Discuss strategy for producing documents; identify who will be involved in which activities; discuss authorship
	8.4	Discuss knowledge translation strategy
**Post-meeting activities**	9^a^	Develop the guidance statement
	9.1	Pilot test the checklist
	10	Develop an explanatory document (E&E)
	11	Develop a publication strategy
	11.1	Consider multiple and simultaneous publications
**Post-publication activities**	12^a^	Seek and deal with feedback and criticism
	13^a^	Encourage guideline endorsement
	14	Support adherence to the guideline
	15	Evaluate the impact of the reporting guidance
	16	Develop Web site
	17	Translate guideline
	18	Update guideline

a Core items (see text).

## Initial Steps

### 1. Identify the Need for a Guideline

Developing a reporting guideline is complex and time consuming, so a compelling rationale is needed. Most reporting guidelines have been developed because researchers are convinced of the need to improve the quality of reporting of a certain type of health research. For some study aspects there may be direct evidence that inadequate reporting is associated with biased reports or harmful consequences. At this early stage, the executive group needs to set out clearly and explicitly their objectives and consider the scope of recommendations. For example, an early decision of the STROBE (STrengthening the Reporting of OBservational studies in Epidemiology) group was to restrict consideration to three main epidemiological study designs [15], leaving the way open to extension guidelines dealing with other designs, such as STREGA (STrengthening the REporting of Genetic Association Studies) [16].

Reporting guideline developers also need to consider whether a need exists to develop a new reporting guideline or to extend or implement an existing guideline.

#### 1.1. Develop new guidance

Developing a new reporting guideline assumes that there is no existing guideline on the topic under consideration. The development of STARD (STAndards for Reporting of Diagnostic accuracy), a reporting guideline for diagnostic accuracy studies, was undertaken because no guidance in this area existed previously and evidence suggested the need for one [17].

#### 1.2. Extend existing guidance

Sometimes there may be the view that an existing broad guideline can usefully be augmented by additional guidance for a specific set of studies. An example of such an extension is the recently published CONSORT (CONsolidated Standards Of Reporting Trials) for nonpharmacological treatments (CONSORT for NPT) [18]. Here we felt that there were specific issues for some types of trials (e.g., surgical) for which sufficient guidance was not offered in the original CONSORT publications. The NPT extension still considers the original 22-item CONSORT checklist as a minimum set of criteria to consider when reporting a randomized controlled trial (RCT), but it extends 11 of these items for further consideration when reporting a trial that has used an NPT intervention.

#### 1.3. Implement existing guidance

Most guidelines are defined by the study aims and methodology, leaving plenty of scope to illustrate their implementation in particular medical specialties. For example, recently a group implemented the CONSORT Statement for behavioral medicine [19], although this implementation was not done in collaboration with the CONSORT Group. Such implementations will generally include requests for additional topic-specific information. The distinction between an extension and an implementation can be unclear.

### 2. Review the Literature

#### 2.1. Identify previous relevant guidance

At this early stage, prospective guideline developers should search for any existing reporting guideline covering all or part of the area being considered. We developed a search strategy to identify and characterize reporting guidelines as part of an ongoing project. In addition to electronic searching, developers are encouraged to search the EQUATOR Network database of reporting guidelines (http://www.equator-network.org). We recommend that reporting guideline developers interested in extending or implementing an existing guideline contact the authors and discuss their plans.

#### 2.2. Seek relevant evidence on the quality of reporting in published research articles

We recommend searching for relevant evidence on the quality of reporting of published research articles within the domain of interest. Such reports provide an initial and important insight into the items to consider for inclusion in an eventual checklist and potential stakeholders to invite to a meeting. However, this literature can be elusive and appear in any journal. A more thorough process, such as a systematic review, would be useful here. The existence of such literature can be most readily ascertained by conducting comprehensive electronic searches of several databases. In preparing for the 2005 QUOROM (QUality Of Reporting Of Meta-analyses) meeting, among other activities we conducted a systematic review of studies that had reviewed the quality of reporting of systematic reviews. To help inform updating of the CONSORT Statement we search the literature every month for new studies of the reporting of randomized trials.

#### 2.3. Identify key information related to the potential sources of bias in relevant studies

For any guideline, both new and revised, there will be key pieces of information that must be included. For example, in a 1993 meeting that ultimately led to the development of the CONSORT Statement, we identified emerging empirical data on the importance of the methods and reporting of allocation concealment when describing the randomization process [20]. The 2010 update of the CONSORT Statement includes new checklist items on identifying any changes to trial outcomes after the trial commenced, and on trial registration. The new item on outcomes follows from empirical evidence [21] indicating that authors frequently do not provide analyses of outcomes in their published papers that were the pre-specified outcomes in their protocols (i.e., selective outcome reporting). The addition of a new trial registration item reflects current journal practice [22] stemming from some researchers hiding results of randomized trials [23].

### 3. Obtain Funding for the Guideline Initiative

To date, no published data inform the costs required to develop a reporting guideline. Even if funding is available, most developers limit their fiscal requests to cover only the main reporting guideline meeting. In our experience, considerable resources are required for pre-meeting activities, such as literature searching and conducting a Delphi exercise, and post-meeting activities, including the need for a small writing group to be able to meet in person, perhaps on several occasions, during the drafting of the guidance and development of an explanation and elaboration (E&E) document.

Depending on the extent of the pre-meeting activities we recommend allocating at least Can$10,000 for them. In our experience the main meeting costs are approximately Can$75,000. This covers the travel and accommodation costs of bringing together 20 to 30 participants. For new guidance in the form of a checklist, with a more detailed and extensive E&E, we recommend at least Can$35,000 for a small writing group to meet several times.

There is no obvious choice as to where reporting guideline developers can seek funding; we are unaware of any granting agency or other group that has a specific remit to provide funding to those interested in improving ways to report health research. Of the 30 respondents to our survey [24], 47% had received funds from a non-profit agency, 17% from the pharmaceutical industry, and 6% from government. We recommend that developers seek funds from all of these sources.

## Pre-meeting Activities

### 4. Identify Participants

Most reporting guidelines have been developed by an international multidisciplinary group involving 22 (median) people [24], although not all may participate in meetings. The expertise of these individuals should reflect the particular guidance under consideration; participants will usually include statisticians, epidemiologists, methodologists, content experts, journal editors, and perhaps consumer representatives. The proportion of content experts needs to be at least a quarter, and perhaps larger depending on the content area under consideration. When developing the herbal extension of the CONSORT Statement we included pharmacognosy experts to provide input on several issues pertinent to reporting herbal interventions.

Well ahead of the proposed meeting date, a list of participants to invite should be developed. The CONSORT executive has typically invited participants in a two-stage process. First, we identify a small group of invitees whose participation we consider essential to hold a meeting. We invite this small group immediately. Once they have accepted our invitation and a date has been settled we invite the remaining participants. We also keep an additional smaller list of people to consider inviting should some second-wave invitees not be able to participate in the meeting. We recommend sending out the meeting invitations well in advance, ideally six months before the meeting date.

### 5. Conduct a Delphi Exercise

Not all potential meeting participants can be invited or will be able to come to the main guideline development meeting. These people can still be engaged in the guideline development process. The CONSORT group has started using a Delphi consensus method [25] to achieve this goal. The Delphi method is a structured process of obtaining information from a group of experts by means of a series of questionnaires, each one refined based on the feedback from respondents on a previous version [10].

To help develop CONSORT for RCT abstracts, we used a three-stage Delphi process. Journal editors, health care professionals, methodologists, clinical trialists, and others with expertise in the reporting of RCTs (*n* = 109) who were known to have an interest in the reporting of randomized trials, the structure of abstracts, or both were invited by e-mail to participate in a Web-based survey and rate the importance of 27 suggested checklist items selected from previous research. During three rounds of the survey, participants were asked about their views on the relative importance of the possible checklist items [25].

### 6. Generate a List of Items for Consideration at the Face-to-Face Meeting

There is no best way to generate the list of items for consideration, which will likely come from a number of sources, such as the Delphi process discussed above (see item 5). For example, the Delphi process for the SPIRIT (Standard Protocol Items for RandomIzed Trials) meeting resulted in 63 nominated items to consider for inclusion in the checklist discussed during the face-to-face consensus meeting. The executive may reduce the initial list of items to a more manageable number for discussion during the face-to-face meeting (see item 8).

### 7. Prepare for the Meeting

#### 7.1. Decide size and duration of the face-to-face meeting

Regardless of the available funding we recommend keeping the size of the meeting to no more than about 30 participants. Larger meetings can lose their spontaneity and limit interaction among the participants, both important attributes of a successful meeting. The CONSORT for herbal medicines meeting was small, involving 16 participants. In contrast, the CONSORT for NPT meeting was large, at 32 participants. Here we were interested in many types of interventions, such as surgery and psychotherapy, requiring a broader number of clinical content experts. It is valuable to have several people who have participated in previous similar meetings.

Reporting guideline meetings that we have been involved in have lasted from one to three days. We recommend a minimum of one day for developing a reporting guideline regardless of whether the guidance is new, an implementation, or an extension.

#### 7.2. Develop meeting logistics

The successful planning and implementation of any reporting guideline meeting requires thorough organization. If funding permits a coordinator should be hired to organize the meeting, including venue selection, meal plans, participant travel and accommodation, and finances.

#### 7.3. Develop meeting agenda

The executive must develop the form and structure of the meeting to ensure adequate time to discuss all of the agenda items. The most important outcome of the face-to-face meeting is an early version of the guidance checklist. We recommend that the following points be considered as part of developing the meeting agenda.

***7.3.1. Consider presentations on relevant background topics, including summary of evidence.*** A useful way to set the stage and maximize participant dialogue is to devote at least a few hours to presentations on topics underpinning the reporting guideline development. Presentations might also be usefully devoted to specific items that are being proposed for the checklist and any relevant empirical evidence.

Ideally, by the conclusion of this session all participants should have up-to-date knowledge about the quality of reporting of the literature at which the guidance is aimed and the evidence relevant to considering the merits of including specific checklist items. Some of this material can be circulated in advance, but it is unlikely that people have time to read extensive materials. A major advantage of the Delphi exercise is that participants can think about some key issues before the meeting.

***7.3.2. Plan to share results of Delphi exercise, if done.*** We have found that a Delphi exercise provides important information when developing a reporting guideline, regardless of whether the guidance is new, an extension, or implementation. The results of a Delphi exercise were presented to participants during the first day of the following three meetings: CONSORT for abstracts, CONSORT for NPT, and SPIRIT.

***7.3.3. Invite session chairs.*** The main meeting will most likely be divided into sessions. While some of the chairs of sessions should be members of the executive group, other participants should also be invited to chair sessions, particularly if they have previous experience developing reporting guidelines and/or chairing meetings. It is essential that chairs have known ability to handle sessions effectively, to ensure that decisions are made.

#### 7.4. Prepare materials to be sent to participants prior to meeting

We recommend sending some materials to participants before the meeting, even though the same materials, and additional ones, should be readily available for each participant during the meeting. Sending the meeting agenda, participant list, one or two papers that might best highlight the quality of reporting of the content area, and the results of any Delphi exercise, if done, is a useful minimum and should be sent to the participants at least a week ahead of the meeting.

#### 7.5. Arrange to record the meeting

The options include audio (and possibly visual) recording of the entire event (this has proved valuable), hiring someone to take comprehensive minutes, or a combination of both. Depending on the meeting agenda, a more focused recording and/or minuting of certain parts of the meeting is another option. As a minimum we recommend comprehensive minuting of all discussions specifically related to the checklist development. Such minuting provides all participants with a record of events and decisions taken during the meeting.

## The Face-to-Face Consensus Meeting Itself

The meeting should follow closely the pre-meeting plans, although timings should be flexible. It is unlikely that all the participants will know each other, so it is helpful for everyone to introduce themselves and indicate the relevance of their particular experience. One of the first tasks of the meeting is to review the objectives and the way the meeting will run, and to clarify any outstanding issues among the participants.

### 8. Present and Discuss Results of Pre-meeting Activities and Relevant Evidence

The substantive meeting begins with any formal presentations of background topics, empirical evidence from the literature, and results of any Delphi process (see item 5 above).

#### 8.1. Discuss the rationale for including items in the checklist

The most detailed and structured discussions at the meeting revolve around which checklist items to include in the guideline; these discussions should focus on information content and not get distracted by seeking agreed wording at this stage. We have always considered the items included in a checklist to be a minimum essential set of items that should be reported, and some discussion of this perspective is valuable before the detailed discussions begin.

The inclusion of each item is ideally supported by empirical evidence, when available. For example, the inclusion of allocation concealment as a checklist item of the original CONSORT Statement was based on an empirical study [20]. There are few such cases. More often there is a consensus that the information is methodologically important to assess in a study; there may also be good evidence that it is frequently not reported. Similarly, evidence and/or conceptual importance in one domain may be transferable from one guideline to another. For example, a specific checklist item on trial registration has been added to the CONSORT 2010 Statement. Similarly, during the 2005 QUOROM meeting we agreed to include a checklist item requesting that authors provide registration information about their systematic review, if available [26]. There may be other reasons for a focused discussion about the inclusion of an item. For example, the CONSORT Statement requests that the study be identified in the title as a randomized trial to aid searching for such studies.

The views expressed in a Delphi exercise, and perhaps also how similar issues were handled in other reporting guidelines, are also important. Ultimately the views of the meeting participants will usually converge, although it may occasionally be necessary to vote on some issues. We recommend considering a classification scheme for selecting items for inclusion in the checklist; we provide an example of this approach that we've used for including items in the CONSORT checklist (see Table 2).

**Table 2:** Classification of categories of items for consideration for inclusion in a reporting guideline checklist, illustrated by some items from the CONSORT checklist.

Item	Item Classification	CONSORT Checklist Number
**Allocation concealment**	An item that is not conducted properly is associated with empirical evidence of bias	9. Mechanism used to implement the random allocation sequence (e.g., sequentially numbered containers), describing any steps taken to conceal the sequence until interventions were assigned
**Blinding**	An item that is not adequately reported is associated with empirical evidence of bias	11a. If done, who was blinded after assignment to interventions (e.g. participants, care providers, those assessing outcomes) and how11b. If relevant, description of the similarity of interventions and procedures
**Sample size**	An item that might not have direct bearing on eliminating bias in the trial's design, but obviously influences its success	7a. How sample size was determined7b. When applicable, explanation of any interim analyses and stopping guidelines
**Numbers analyzed**	An item that reflects upon trial conduct and impacts upon internal and external validity	16. For each group, number of participants (denominator) included in each analysis and whether the analysis was by original assigned groups
**Outcomes and estimation**	An item that reflects the crucial trial results	17a. For each primary and secondary outcome, results for each group, and the estimated effect size and its precision (e.g., 95% confidence interval)17b. For binary outcomes, presentation of both absolute and relative effect sizes is recommended
**Participants**	An item that is essential for external validity (generalisability, applicability)	4a. Eligibility criteria for participants4b. Settings and locations where the data were collected
**Interpretation**	An item that aids in the interpretation of the results	22. Interpretation consistent with results, balancing benefits and harms, and considering other relevant evidence

#### 8.2. Discuss the development of a flow diagram

The majority of reporting guidelines have been limited to developing a checklist [24]; a few groups have also developed a flow diagram, the most well known of which is the CONSORT diagram. We recommend that the meeting agenda include discussion of the possibility of developing a diagram and, if appropriate, consideration of its content.

#### 8.3. Discuss strategy for producing documents; identify who will be involved in which activities; discuss authorship

The most important deliverable is the final reporting guideline. Commonly this will be in the form of a journal article, written after the meeting that introduces the checklist (and flow diagram) and summarises the processes used to develop it. Some groups such as CONSORT refer to this document as a “Statement” to distinguish it from other types of publication. A detailed explanatory paper, an E&E, may also need consideration. Sufficient time needs to be included in the meeting agenda to discuss these activities. Developing an E&E is very time consuming, and will require further input from the meeting participants (see item 10). To accomplish this task we have often asked meeting participants, all of whom would be considered for authorship, to volunteer to help draft particular sections. Time needs to be set aside to discuss these issues during the meeting and the authorship model needs to be agreed upon.

#### 8.4. Discuss knowledge translation strategy

One of the last major sessions of the meeting should be devoted to issues pertaining to disseminating the reporting guideline. There are several points to consider here, especially a publication strategy. Since the simultaneous publication in three journals of the 2001 CONSORT Statement, several reporting guidelines have been published in multiple journals. Ultimately reporting guideline developers want to positively influence the reporting of health research and will need to consider how best to ask journals and editorial groups (such as the International Committee of Medical Journal Editors), for help achieving these goals. To address these issues adequately the PRISMA (Preferred Reporting Items for Systematic reviews and Meta-Analyses) Group formed a three-member dissemination committee. How to maximize journal endorsement and adherence to the reporting guideline can also be discussed during the meeting. Additional points for discussion can include: whether and how the guidance will be formally evaluated; how any criticism will be handled; and whether to create a Web site and what it might contain.

## Post-meeting Activities

Together with drafting and finalizing guideline documents the reporting guideline developers need to consider their implementation strategy, including publication issues (open access, copyright, peer review, and multiple and possibly simultaneous publications), website development, and seeking or monitoring endorsement of and adherence to the reporting guideline.

### 9. Develop the Guidance Statement

We recommend that initial efforts be focused on drafting the checklist for the proposed reporting guideline. Efforts aimed at extending or implementing existing reporting guidelines should make very clear which parts of their checklist remain the same as the original and which items have been modified or added. The process of developing the checklist is very likely to require several iterations. Initially, this entails taking the discussions from the face-to-face meeting (item 8.1 above) and crafting each item into a crisply and unambiguously worded checklist item. The most appropriate order for the checklist items also needs consideration.

For a new reporting guideline we recommend a short document of about 2,000 words reporting on the rationale for developing the guidance and the development process, including a brief description of the meeting and participants involved. The article will include the checklist and flow diagram, if developed. In our experience drafting the checklist and article is best done by a small writing group made up primarily of members of the executive team and perhaps one or two others. This group will usually constitute the guideline authors on behalf of the reporting guideline group, although group authorship may be preferred. For extending an existing reporting guideline a similar approach is recommended. Whether a new guideline is being developed or an existing one is being extended, the full guideline group will need to sign off on the document's content prior to journal submission.

#### 9.1. Pilot test the checklist

Pilot testing the checklist and flow diagram is worth considering. During the development of the QUOROM Statement, two members of the executive group independently asked two separate sets of students taking a systematic review course for their feedback on the checklist that was used to help report their systematic review. Their comments were incorporated into the checklist revisions.

### 10. Develop an Explanatory Document (E&E)

To date most reporting guidelines have not been accompanied by a detailed justification and explanation of the recommendations (e.g., STRICTA [STandards for Reporting Interventions in Controlled Trials of Acupuncture]). However, we believe that it is vitally important to provide an explanation of the rationale and evidence for a guideline item's inclusion and an elaboration on the details of an item, hence the title Explanation and Elaboration, or E&E, adopted by CONSORT and several other groups.

While there will undoubtedly be an urge to complete all of the guideline revisions rapidly and submit the paper for publication consideration, we recommend holding off doing so until an E&E has been prepared (see Box 1). The E&E will be considerably longer than the guideline statement (probably 10,000–20,000 words). As with the short guideline statement (see item 13) the full guideline group will need to sign off on the E&E's content before journal submission.

**Box 1. The Rationale for Developing an Explanatory Document (E&E) to Support a Reporting Guideline**

A reporting guideline gives a set of recommendations regarding the information that should be included in the report of a research study. It may also include guidance on how some information may best be presented (e.g., in a Table, or as absolute numbers).

Guideline developers aim to persuade editors and authors of the importance of adhering to their recommendations. To that end, simply providing a list of requirements, often as a checklist, is unlikely to be sufficient. Such a terse presentation may seem to make unsupported, even dogmatic statements without clear support, even when supported by relevant references to other publications. That concern underpinned the decision of the CONSORT Group to accompany the revised CONSORT Statement [44] with a detailed explanatory document, which was named “Explanation and Elaboration” (E&E) [45].

The E&E was intended to provide detailed rationales for all of the items in the CONSORT checklist and flow diagram. For each item, the paper included (a) an example of good reporting from a published paper, (b) the scientific background and rationale for including that information in a published article, (c) empirical evidence of bias associated with the way that aspect of a study is conducted or reported, and (d) any evidence relating to the extent of inadequate reporting of that information. In addition, some fundamental concepts that underpinned several of the items were discussed in boxes.

The novel format of the CONSORT E&E was emulated for several later reporting guidelines—STARD, STROBE, and PRISMA [46]–[48]. The broad format was retained with little modification, although the later examples have included rather more explanatory boxes; for example, the STROBE paper has eight boxes, including those addressing missing data, bias, interaction (effect modification), and how to use the paper. For some guidelines, notably some extensions of the CONSORT Statement, the explanatory information has been included with the reporting recommendations in a single article (e.g., CONSORT cluster [49], NPT [18]).

We feel that simultaneous publication of the statement along with an E&E is the most important way to disseminate the work of the reporting guideline group. For limited extensions a single article may best cover both roles, as was done, for example, for CONSORT for herbal interventions [27] and CONSORT for NPT [18].

### 11. Develop a Publication Strategy

We recommend that guideline developers negotiate a copyright agreement to retain the rights of the contents of the statement and related documents, such as the E&E. We have not found journals to be resistant to this idea. Similarly, we recommend developers to negotiate being able to put the reporting guideline on a dedicated website.

#### 11.1. Consider multiple and simultaneous publications

Starting with the revised CONSORT Statement in 2001, several developers have published their guidance in multiple journals simultaneously to enhance the uptake and dissemination of their reporting guideline (STARD, STROBE, STREGA, PRISMA). For example, the STARD Statement for reporting diagnostic accuracy studies is relevant to a broad spectrum of potential users, from clinical chemists to radiologists, all of whom might not be aware of each other's literature. Publishing in a radiology journal [28] and a clinical chemistry journal [29] likely increased the reach and potential influence of the guidance.

Our experience is that the journal submission and peer review processes for multiple simultaneous publications can be exhausting. Some steps can reduce the burden. For example, the guideline author group might want to nominate an author as corresponding author to facilitate the coordination process with the various journals. Before submission it might be useful for the reporting guideline authors to discuss with prospective journals their interest in publishing the guidance and the need for all journals to agree a common text. When submitting the final manuscript the journals can be asked about their willingness to have a common set of peer reviewers. Such a move enables the reporting guideline developers to respond to a single set of peer reviewers rather than separately to peer reviewers from each journal. Similarly, to ensure consistency across journals we believe that copy editing should be limited to spelling, layout, and punctuation, ensuring identical text across journals. This might be best achieved by having one lead journal take care of these issues and circulating the copy edited version across the other journals publishing the article. The PRISMA Statement, recently published in five journals [30]–[34], used these approaches; one journal volunteered to coordinate the whole editorial process and the other journals used their peer reviews and copyediting, thus making it more efficient for the developers and journals alike.

## Post-publication Activities

### 12. Seek and Deal with Feedback and Criticism

It is important to seek feedback and criticism from all stakeholders regarding the reporting guideline, and we have outlined several stages by which this can be achieved in the development process. We recommend that guideline developers also encourage feedback at any time after publication, either directly or via the related Web site. Constructive criticism can help improve the reporting guideline if an update is prepared (see item 18, below). After the CONSORT Statement was originally published, Meinert [35] provided valuable suggestions on ways to improve the flow diagram and the clarity of some of the terminology initially used. These recommendations were incorporated into the 2001 revision of the CONSORT Statement.

### 13. Encourage Guideline Endorsement

Some journals, those whose editors initially were involved in the guideline development, will be eager to support the use of the reporting guideline. This support can be most readily achieved when a journal endorses the reporting guideline. However, some journals have used inconsistent language to describe their endorsement [36]–[38], and this vagueness likely diminishes serious efforts to improve the quality of reporting within journals.

When journals want to endorse a reporting guideline we recommend that they use strong, clear language of their expectations of authors, and include this information in their Instructions to Authors. For example, BioMed Central, the publisher of 199 open access journals, states, “We recommend authors refer to the EQUATOR network website for further information on the available reporting guidelines for health research, and the MIBBI Portal for prescriptive checklists for reporting biological and biomedical research where applicable. Authors are requested to make use of these when drafting their manuscript and peer reviewers will also be asked to refer to these checklists when evaluating these studies” (http://www.biomedcentral.com/bmcdermatol/ifora/).

Reporting guideline developers might also consider developing some brief text to help journals introduce their guidance to authors, incorporating the issues discussed above. We also recommend that when a journal endorses a reporting guideline they notify the guidance developers. This will help the developers to document and track all endorsements.

### 14. Support Adherence to the Guideline

Adherence is not part of developing a reporting guideline. However, it is central to whether reporting guidelines have their intended impact. We recommend that guideline developers consider issues of adherence regarding their reporting guideline, whether it is a new one or an extension or implementation of an existing one.

Journal endorsement, while encouraging for guideline developers, needs to be accompanied by a clear statement of how the journal expects authors to use the guideline and what level of adherence is required (e.g., authors of reports of randomized trials must submit a completed CONSORT checklist along with their submission). We recommend that journals consider ways to maximise adherence to reporting guidelines, such as by asking authors to submit completed checklists and by asking peer reviewers to use them as part of their review.

### 15. Evaluate the Impact of the Reporting Guidance

We recommend assessing the impact of any reporting guideline. Unfortunately, few guidelines appear to have been evaluated to date [24]. Although the guideline developers will likely want to evaluate their guidance, and we support such enthusiasm, other researchers should be also encouraged to conduct these assessments. There are several ways to design and carry out such an evaluation. Results from the systematic review on CONSORT evaluations suggest there is considerable room to improve the designs of these evaluations [39].

### 16. Develop Web Site

Another implementation strategy is to create a Web site dedicated to the reporting guideline. It makes the most sense to develop the Web site before publication so that the address can be included in the published articles. As a minimum we recommend including on the Web site the reporting guideline checklist, statement, ancillary documents such as an E&E, and any translated versions of the guideline. Also, the list of participants and funders should be included. The checklist and diagram should be available as both PDF and DOC files. Related unpublished documents can be published here too. The Web site is also a useful venue for alerting readers about emerging issues related to the guidance and perhaps inviting comments and discussion. We also recommend including a news section where new information about the guidance can be posted. Unfortunately, the lack of funding may impede such valuable additions.

We recommend including an endorser section for the names of journals and editorial groups endorsing the reporting guideline. The journals' Instructions to Authors can be linked to their endorsement.

The Web site can also provide policy documents regarding the use of the guidance. Issues pertaining to copyright, use of documents and, if relevant, the permitted use of the guidance logo if there is one, can be included in this section. Finally, we encourage guideline developers to consider linking their guideline with the EQUATOR Network (http://www.equator-network.org).

### 17. Translate Guideline

After publication of the reporting guideline it is possible that other researchers will want to translate the reporting guideline into different languages. The developers should seek to be actively involved to assure themselves that all translations are completed appropriately using robust methods including back translation.

### 18. Update Guideline

As yet few reporting guidelines have been updated, although most (83%) guideline developers recognize the need to do so [24]. The trigger to update is complex and likely similar to other areas of research [40]. Chief among these is the body of emerging literature to inform the currency of the checklist. If this literature is large and includes policy or empirical evidence it is likely important to consider updating. For example, recent empirical evidence [21] on outcome reporting bias led the CONSORT Group, when preparing CONSORT 2010, to add a checklist item specifically asking authors to describe any changes made to their study outcomes between the protocol and final analysis.

We recommend regular consideration of whether or not to update a reporting guideline and that reporting guideline groups maintain an executive group that can help make such decisions. Although it is possible to make frequent (e.g., annual) small updates, we believe this is not wise and it may even be counterproductive. Making occasional major updates, with clear version numbers, seems to be a better approach. We convened a one-day meeting to update the CONSORT Statement and two-day meeting to update QUOROM (subsequently published as the PRISMA Statement).

We assume that updating an existing reporting guideline will be completed by essentially the same team that initially developed the guidance, although some of the membership may change over time. That has been our experience from the CONSORT Statement updates. We also assume that several components of the initial guideline development process are still in place. For example, for updating the guideline, the developers would need to update their existing literature searches.

When considering an update to an existing reporting guideline, developers should give serious consideration to the seven essential items (denoted with an †) in Table 1. The development of an E&E deserves serious consideration when updating a reporting guideline if none existed before. If there is one, then it too will need updating (see Box 1). It is helpful to include in the publication a list of what was changed and why.

We also encourage reporting guideline developers to seriously consider how best to handle numbering and dates of updates. For example, the original version of CONSORT did not number the checklist items. We introduced numbering into the 2001 version, which meant that we have had to be careful in numbering new checklist items in the latest (2010) update. We have modified the numbering slightly. Updating a reporting guideline will influence checklist items of any published extensions and/or implementations, and due consideration is required as to how best to handle these issues.

## Discussion

We hope this guidance on how to develop a reporting guideline, including an 18-step checklist, will fill a gap in the literature and be of help to potential and practicing developers. While some of the items are optional, there is a core set of steps we believe necessary to ensure adequate development of a reporting guideline. In the future, the EQUATOR Network might consider providing an appraisal grade for the reporting guidelines included on their database, reflecting the robustness of the guideline development process. We also encourage prospective guideline developers to contact the EQUATOR Network team to inform them about their work; this might prevent duplication of effort.

We will make the 18-step checklist available on the EQUATOR Network Web site. We will continue to monitor the literature to help maintain the guidance, particularly the checklist. Furthermore, we will annually review the need to update the checklist.

While there is increasing evidence that use of reporting guidelines is associated with improvements in the quality of reporting health research [37],[39],[41],[42], there is a growing anecdotal belief that reporting guidelines indirectly have a positive impact on how researchers design and conduct their research [43]. More formal research is required to substantiate these claims. Beyond the possible benefit in the design and conduct of health research, reporting guidelines are now starting to be used as an adjunct in developing educational courses in the design and conduct of health research.

Reporting guidelines are currently focused at the end of the knowledge generation cycle. However, we believe that investigators would benefit from the knowledge of key principles of health research reporting and relevant guidelines at the beginning of their research, having the end in mind. Indeed, guidelines are possibly equally useful earlier on in this process, and some granting agencies have acted upon this concept. The SPIRIT initiative is aimed at providing guidance for writing protocols of RCTs. Similarly, the UK National Institute of Health Research developed a research process flowchart (http://www.rdinfo.org.uk/) to guide researchers in how to start developing a research project. The flowchart includes reference to reporting guidelines and encourages researchers to “consult a relevant guideline in the early stages of research planning.”

Given the broad range of health research now covered by reporting guidelines, funders might start to require prospective applicants to use some parts of an “approved” reporting guideline when developing their research application and to include a completed checklist as part of this process. This might increase the overall quality of the applications to funders while increasing the potential return on their investment by emphasizing the importance of reporting much earlier in the knowledge generation cycle.

Reporting health research in a complete, accurate, transparent, and timely manner is a shared responsibility of all stakeholders involved in research funding, conduct, and publication. High-quality research reports contribute to more efficient translations of new research findings into clinical practice and help advance scientific knowledge and patient care. We will all benefit from these collective efforts.

## Abbreviations

CONSORT: CONsolidated Standards Of Reporting Trials
CONSORT for NPT: CONsolidated Standards Of Reporting Trials for Non-Pharmacological Treatment interventions
E&E: Explanation and Elaboration
EQUATOR: Enhancing the QUAlity and Transparency Of health Research
PRISMA: Preferred Reporting Items for Systematic reviews and Meta-Analyses
QUOROM: QUality Of Reporting Of Meta-analyses
RCT: randomized controlled trial
SPIRIT: Standard Protocol Items for RandomIzed Trials
STARD: STAndards for Reporting of Diagnostic accuracy
STREGA: STrengthening the REporting of Genetic Association Studies
STRICTA: STandards for Reporting Interventions in Controlled Trials of Acupuncture
STROBE: STrengthening the Reporting of OBservational studies in Epidemiology
